# Sea-Based Infrared Scene Interpretation by Background Type Classification and Coastal Region Detection for Small Target Detection

**DOI:** 10.3390/s150924487

**Published:** 2015-09-23

**Authors:** Sungho Kim

**Affiliations:** Department of Electronic Engineering, Yeungnam University, 280 Daehak-Ro, Gyeongsan, Gyeongbuk 38541, Korea; E-Mai: sunghokim@ynu.ac.kr; Tel.: +82-538-103-530

**Keywords:** infrared scene interpretation, background type, coastal region detection, line-of-sight, horizontal line, region-dependent detector

## Abstract

Sea-based infrared search and track (IRST) is important for homeland security by detecting missiles and asymmetric boats. This paper proposes a novel scheme to interpret various infrared scenes by classifying the infrared background types and detecting the coastal regions in omni-directional images. The background type or region-selective small infrared target detector should be deployed to maximize the detection rate and to minimize the number of false alarms. A spatial filter-based small target detector is suitable for identifying stationary incoming targets in remote sea areas with sky only. Many false detections can occur if there is an image sector containing a coastal region, due to ground clutter and the difficulty in finding true targets using the same spatial filter-based detector. A temporal filter-based detector was used to handle these problems. Therefore, the scene type and coastal region information is critical to the success of IRST in real-world applications. In this paper, the infrared scene type was determined using the relationships between the sensor line-of-sight (LOS) and a horizontal line in an image. The proposed coastal region detector can be activated if the background type of the probing sector is determined to be a coastal region. Coastal regions can be detected by fusing the region map and curve map. The experimental results on real infrared images highlight the feasibility of the proposed sea-based scene interpretation. In addition, the effects of the proposed scheme were analyzed further by applying region-adaptive small target detection.

## 1. Introduction

Infrared search and track (IRST) is an important technology for protecting the homeland from sea skimming missiles, multiple rocket launchers (MRLs), coastal guns and asymmetric boats [1]. Although the real detection range of sea-based IRST is approximately 30 km (assuming a target height of 100 m and a sensor height of 30 m), IRST is adopted frequently in ships to complement radar because it can provide a high precision target location [2]. The main purpose of IRST is to achieve a high detection rate with a low false alarm rate. Several spatial filter-based [3,4,5] or temporal filter-based [6,7,8,9] small infrared target detection methods have been proposed. Spatial filter-based methods use a background estimation and subtraction approach. The background can be obtained using the least mean square filter [3], median filter [10], mean filter [4] or morphological filter [5]. Temporal filter-based methods use motion information to extract the targets from the background. The motion can be extracted using track-before-detect (TBD) [6], dynamic programming [11] and temporal profiles [7,9,12].

**Figure 1:**
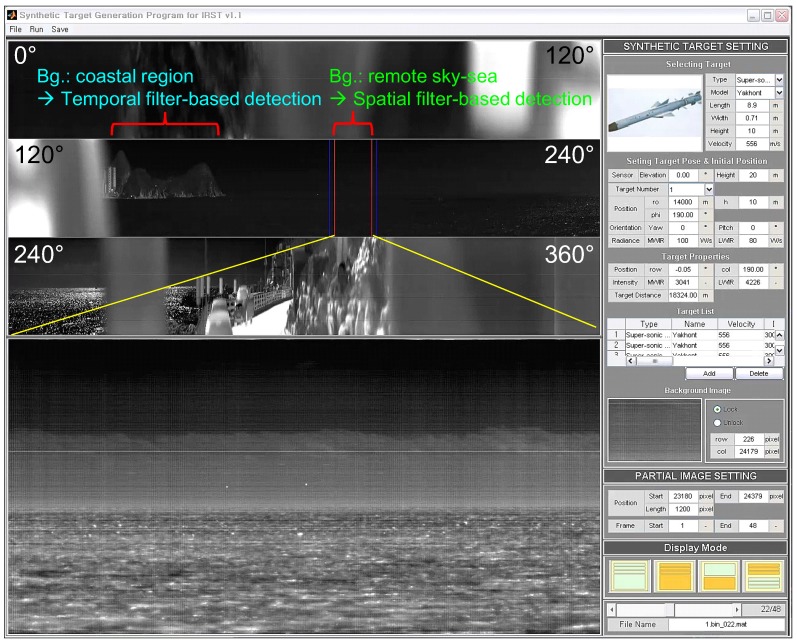
Motivation for the background classification and coastal region detection for sea-based infrared search and track: different types of small infrared target detection methods should be applied depending on the background to maximize the detection rate and to minimize the false alarm rate.

These small target detection approaches show good performance in their specific application domains. On the other hand, most of these small target detection algorithms use infrared images with a narrow field of view (NFOV) for specific backgrounds. According to the operational concept of the IRST system, omni-directional infrared images should be acquired and used for surveillance with both long-range capability and coastal environments [13]. In this paper, omni-directional infrared imaging systems refer to a $360^{\circ }$ field of view (FOV), not fish eye or wide FOV cameras. A parabolic mirror system in front of an infrared camera can provide $360^{\circ }$ FOV with reduced image resolution, which is not suitable for a long-range surveillance system [14]. Although omni-directional infrared imaging systems, such as Veille Air-Mer Panoramique Infrarouge (VAMPIR) [15] and Advanced Reliable Third Generation Naval IRST (ARTEMIS) [16,17], have been developed and deployed in several countries, there are almost no reports related to small target detection for omni-directional IRST systems. Only Weihua *et al*. have proposed a simple temporal difference-based detection method for aerial moving targets [18]. Figure 1 presents an omni-directional image, where the three rows represent a wide field of view ($120^{\circ }$ each), and the fourth image shows an enlarged image for the specific sector. The panoramic image contains both sky-sea background and coastal regions.

**Figure 2:**
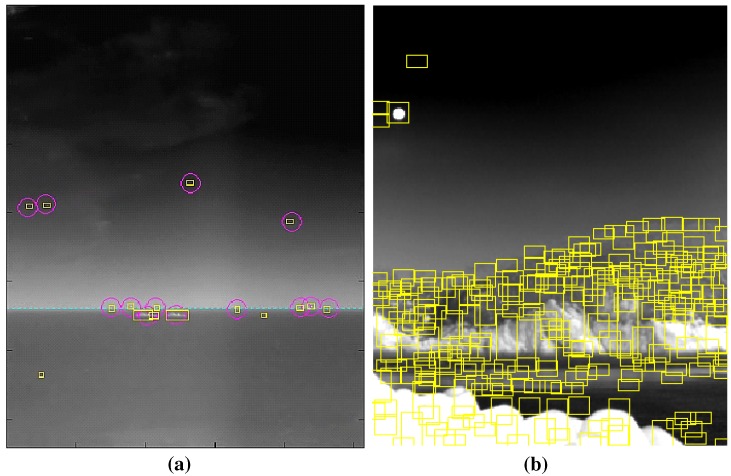
Comparison of spatial filter-based infrared target detection for different types of background: (**a**) sky-sea; (**b**) coast region. The yellow squares represent the detected target regions.

Different types of small infrared target detection methods are needed to satisfy both the detection rate and false alarm rate. The performance of the detection rate (DR) and false alarm rate (FAR) should be compared on the same public database. On the other hand, there is no public infrared database for small target detection problems. According to the authors’ experience, long-range targets normally exist around a horizontal line and are almost stationary with a very low signal-to-noise ratio. As shown in Figure 2a, spatial filter-based background estimation and subtraction methods can detect remote targets quite well [5,19,20,21]. If, however, the same method is applied to coastal regions, many false detections are produced due to ground clutter, as shown in Figure 2b. Normally, coastal regions are relatively close to a ship. This means that the target movement can be detected easily in an infrared image using temporal filters. Therefore, the detection of coastal regions is very important to the success of IRST operation in a real-world scenario by scene-dependent target detection.

Although many studies have examined small infrared target detection, there are no reports related to infrared coastal region detection, except for Kim *et al.*’s initial work [22]. The method used a region segmentation and sky-coast line detection scheme. Kim *et al.* attempted to find the borderlines between the sky and coast regions using region contrast and statistical properties. In this study, the method was extended to an infrared scene interpretation by incorporating a background type classification with coastal region detection. Therefore, the contributions of this paper can be summarized as follows. First, a novel sea-based infrared scene interpretation method is proposed for the first time by cascading the background type classification and coastal region detection. Second, a novel sea-based infrared scene classification method is proposed using the relationships between the geometric horizon and the image horizon. Third, a new coastal region extraction method is proposed by the horizon-guided image fusion of the region map and the curve map. Fourth, the infrared scene interpretation method was validated using real infrared images. Finally, the effects of the infrared scene interpretation were found by applying a scene-dependent small target detection strategy for the synthetic database and real target sequence. Section 2 introduces the overall structure of the proposed infrared scene interpretation method, where the scene type classification method is explained. In addition, a precise coastal region detection method is presented for the coastal background type. In Section 3, the experimental results on real scenes with synthetic targets validate the power of the proposed architecture. Section 4 concludes and discusses the paper.

## 2. Proposed Infrared Scene Interpretation System

**Figure 3:**
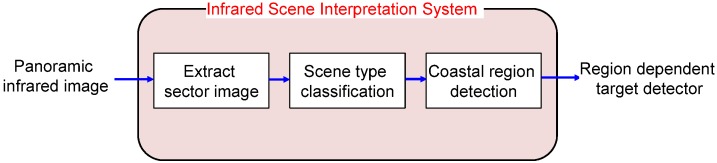
Proposed infrared scene interpretation system.

The key components of an omni-directional infrared search and track are sector image extraction, scene type classification and coastal region detection, as shown in Figure 3. An IRST sensor scans the scenes continuously, and each image sector containing a $10^{\circ }$ field of view is extracted and transferred to the target processing board. The next step is to determine the types of sector images. The scene types are sky-sea, remote coast, homogeneous near coast and cluttered near coast. The scene classification is based on the relationship between the geometric horizon position and the image horizon position. The proposed coastal region detector is activated iff the scene type contains a coastal region. Information about the coastal region is used in the following small target detector.

### 2.1. Properties of the Sea-Based IRST Background

**Figure 4:**
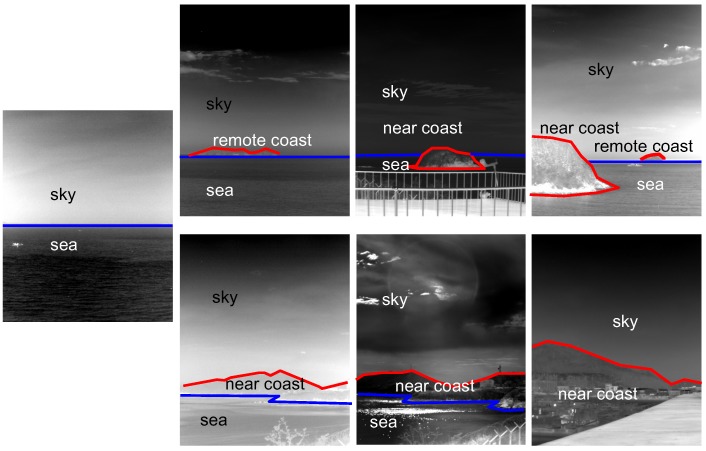
Proposed infrared scene interpretation system.

Sea-based IRST background images should be observed and analyzed carefully to obtain important domain knowledge. Figure 4 shows representative images used in the sea-based IRST system. Most of the background consists of a sky-sea region, sky-coast-sea or sky-near coast. Direct observations lead to three properties.

- The shapes of the sky, coast and sea regions are wide, because the imaging view point is slanted.
- The order of the background is predictable, such as sky-sea, sky-coast-sea and sky-coast. The reverse order is not permitted.
- A lower coast region generally occludes other remote regions due to the geometry of the camera projection.

**Figure 5:**
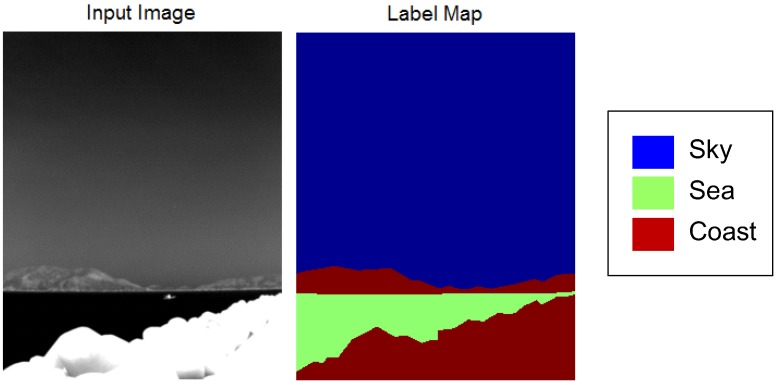
Example of the prepared test DB for a quantitative inspection.

The next inspection is based on quantitative measurements of the temperature and texture distributions. Forty-one infrared background images were prepared and labeled. Figure 5 provides an example of an infrared background image and the corresponding label map consisting of sky, sea and coast regions.

**Figure 6:**
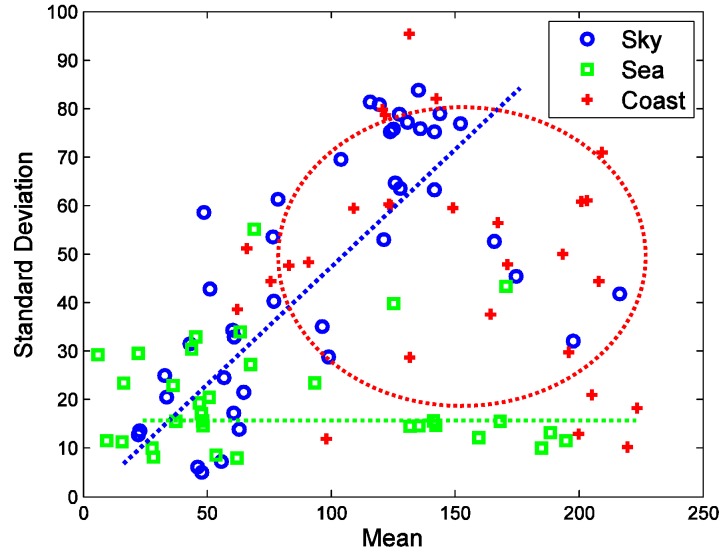
Distribution of the thermal average intensity and standard deviation for each region.

Figure 6 summarizes the distribution of the thermal average intensity and standard deviation. The sky region shows a wide thermal average intensity with a relatively low standard deviation. On the other hand, the sky region shows a proportional relationship between thermal intensity and the standard deviation. This originates from clear sky and warm clouds. Clear sky has a very low temperature with a low standard deviation. Warm clouds have a high temperature and a high standard deviation around the cloud edge. The coast region does not have any correlation between the thermal average intensity and the standard deviation. The coast region normally has a wide intensity and a wide standard deviation owing to its complex nature.

**Figure 7:**
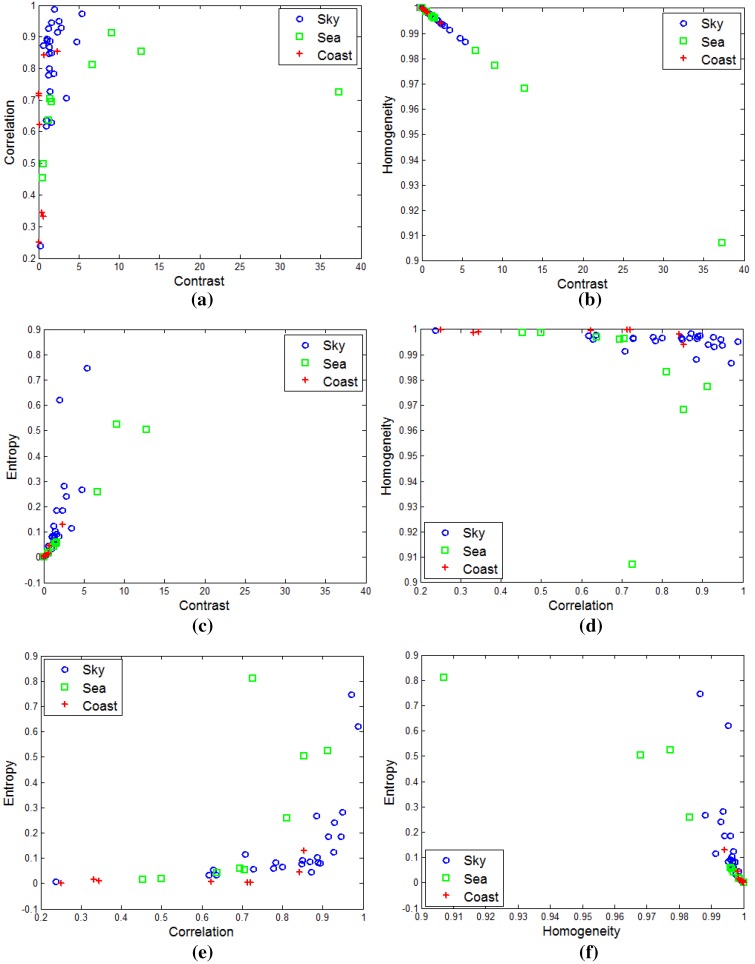
Distributions of the gray level co-occurrence matrix (GLCM)-based thermal texture for each region: (**a**) contrast *vs.* correlation; (**b**) contrast *vs.* homogeneity; (**c**) contrast *vs.* entropy; (**d**) correlation *vs.* homogeneity; (**e**) correlation *vs.* entropy; (**f**) homogeneity *vs.* entropy.

A quantitative inspection of the thermal texture can be estimated from the gray level co-occurrence matrix (GLCM) proposed by Blanton and Barner [23]. The GLCM ($P_{d}\left(i,j\right)=n_{ij}$) can be obtained by counting ($n_{ij}$) the neighboring pixels with the specific gray values (i,j) in a probing image. The representative texture features are contrast, correlation, homogeneity and entropy [23]. Figure 7 shows the distribution of the GLCM-based texture using different tools for the sky, sea and coastal regions. The sea-based IRST images show no distinctive texture features. Most distributions are mixed and concentrated on the corners. Therefore, texture-based infrared image segmentation is not a suitable approach.

### 2.2. Infrared Background Type Classification

**Figure 8:**
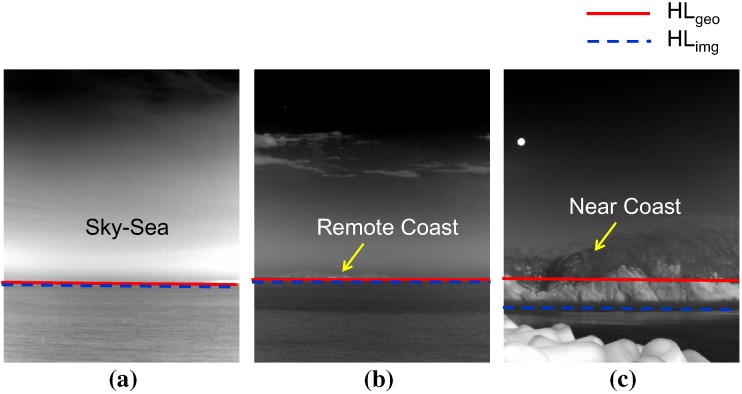
Horizon observation of the sea-based infrared images and background types: (**a**) normal sky-sea; (**b**) remote coast; (**c**) near coast background.

The sea-based infrared image types are normal sky-sea, remote coast and near coast background, as shown in Figure 8, according to the observation. The problem is how to classify the infrared background type using a simple, but robust method. The first idea is to use the horizontal information predicted by the sensor pose and the observed horizon position. As shown in Figure 8a,b, the horizon position (red solid line) predicted by sensor pose [24] is identical to the observed horizon (blue dotted line) for remote sea. In a near coast environment, the real horizontal line deviates from the predicted horizontal line, as shown in Figure 8c. The second key idea is to use the number of detection results to estimate the clutter density of the probing scene. Normally, a large number of false alarms is prohibited in an IRST system. On the other hand, the number of false detections can provide useful information regarding the coast types, as shown in Figure 2.

**Figure 9:**
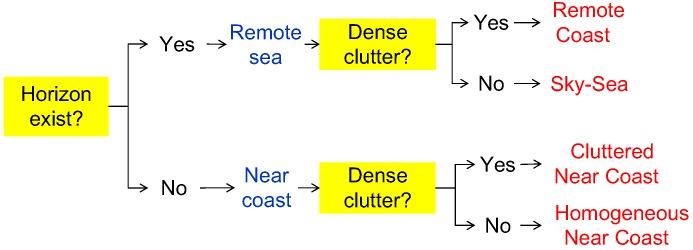
Proposed infrared scene classification method using the horizon and clutter density cues.

Figure 9 summarizes the proposed infrared scene classification method. The remote sea and near coast are determined depending on the existence of the horizon. In the remote sea case, the scene is classified as remote coast if there is dense clutter. Similarly, the scene is determined to be a cluttered near coast if there is dense clutter in the near coast.

The first criterion is called the horizon evidence function ($H_{evid}$) and is defined as Equation (1).

(1) $$H_{evid}\left(H_{geo},H_{img}\right)=\left\{\begin{array}{cc} 1 & \left|H_{geo}-H_{img}\right|<\delta \\ 0 & \text{else} \end{array}\right.$$

where $H_{geo}$ denotes the sensor pose-based horizon prediction in the image and $H_{img}$ denotes the horizon position measured from the infrared image. If the difference margin is smaller than δ (normally four pixels), the real horizon can be confirmed to exist. $H_{geo}$ was derived in [24] and is briefly introduced in this paper. Consider the IRST sensor height *h*, elevation angle α, field of view β and Earth radius *R*. The angle of the calculated horizontal line in an image can be found using Equation (2) assuming α=0.

(2) $$\begin{array}{c} \theta_{H}=-cos^{-1}\left(\frac{R}{R+h}\right) \end{array}$$

If the elevation angle is considered, the amount of sky region ($\theta_{sky}$) can be calculated using Equation (3).

(3) $$\theta_{sky}=\left\{\begin{array}{cc} 0 & \text{if}\;\alpha <\theta_{H}-\beta /2\\ \beta & \text{if}\;\alpha >\theta_{H}+\beta /2\\ \alpha -\theta_{H}+\beta /2 & \text{else} \end{array}\right.$$

The geometrically-predicted horizontal line ($H_{geo}$) can be found using Equation (4). The angle of the sea region ($\theta_{sea}$) is just $\beta -\theta_{sky}$.

(4) $$\begin{array}{c} H_{geo}\left[pixel\right]=ImageHeight*\frac{tan(\theta_{sky})}{tan\left(\theta_{sky}\right)+tan\left(\theta_{sea}\right)} \end{array}$$

**Figure 10:**
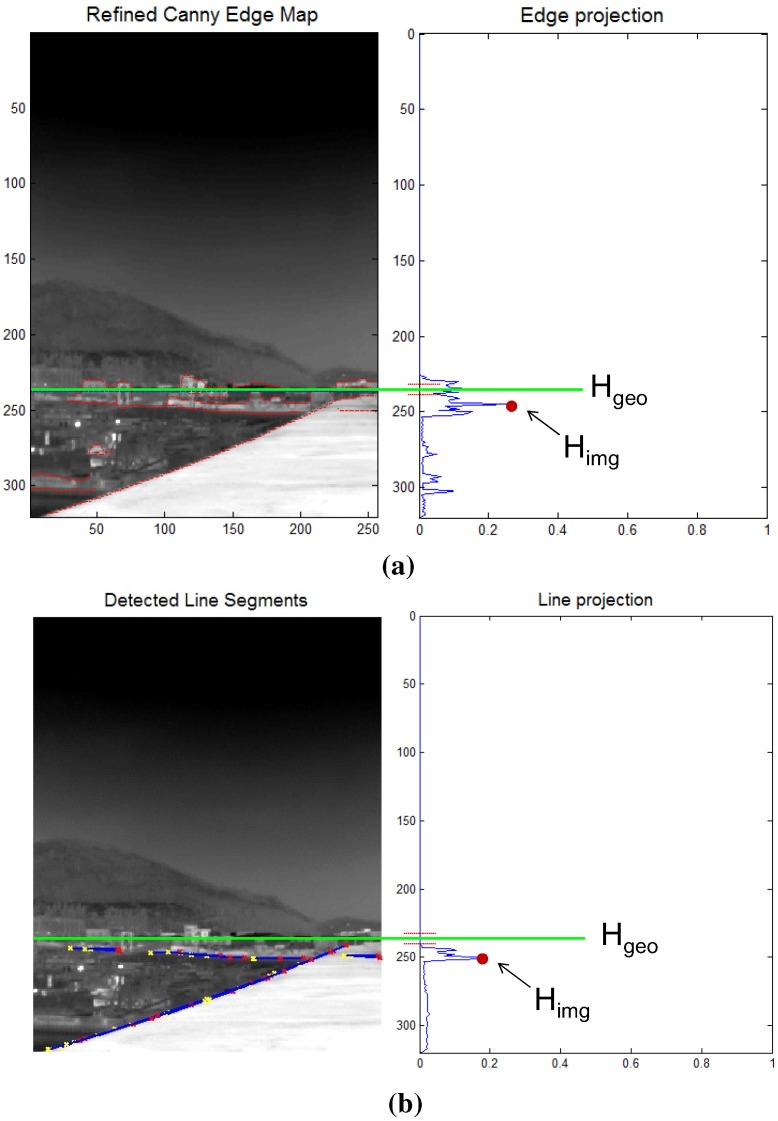
Estimation of the horizon in image ($H_{img}$) using the projection method in the near coast region: (**a**) edge map-based measurement; (**b**) line map-based measurement.

How can a horizon position ($H_{img}$) be found in an image? The camera roll angle is assumed to be zero to simplify the problem. The key idea of the $H_{img}$ estimation is to accumulate horizon-like pixel information (EM(i,j)) along each image row and to find a row index (*i*) that maximizes the horizon density using Equation (5). The probing image size is M×N with a row index *i* and column index *j*. The edge map should be selected carefully to achieve the robustness of a horizon estimation in an image. If a well-known Canny edge detector [25] is applied, as shown in Figure 10a, the accumulated edge map shows an erroneous distribution of horizon density. Instead of a simple edge map, a line map is used, as shown in Figure 10b. The line segments are detected by applying a Hough transform [26] to the edge map. Therefore, the remote sea and near coast can be classified using Equation (1), Equation (4) and Equation (5).

(5) $$\begin{array}{c} H_{img}=\underset{i}{max}\left(\sum_{j=1}^{N}EM\left(i,j\right)\right) \end{array}$$

**Figure 11:**
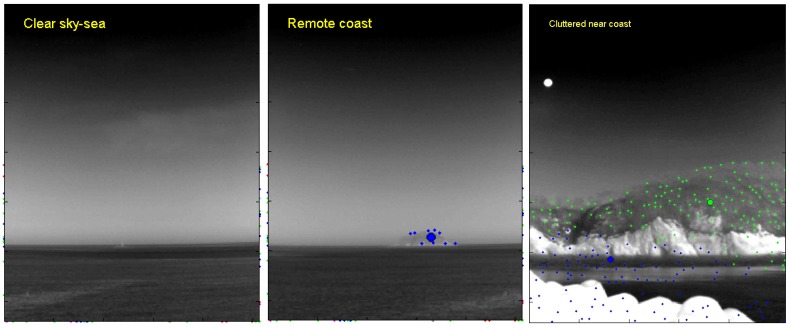
Examples of scene classification using the proposed background type classification.

The second criteria is the clutter density to further classify the infrared background. The clutter density provides useful scene information on the coast types, as shown in the infrared scene type diagram (see Figure 9). The clutter density can be measured directly from the infrared images, such as the standard deviation [27]. On the other hand, the low level intensity-based variance shows very different behavior compared to coastal clutter. Therefore, this paper proposes a new clutter density ($D_{clutter}$) measurement, as expressed in Equation (6), where $N_{pre-detect}$ denotes a spatial filter-based small target detector with a pre-detection threshold in the hysteresis detector [24]. Area represents the area (pixels) occupied by pre-detection. If the density is larger than 5 clutters/(100 × 100 pixels), the scene is classified as cluttered coast. The mean-shift clustering method [28] is applied to the pre-detection results to estimate the area covered by clutter. Figure 11 presents representative examples of an infrared scene classification using the proposed rule-based algorithm with the horizon existence and clutter density function. Infrared images of the remote sky-sea, remote coast and cluttered near coast are classified correctly.

(6) $$\begin{array}{c} D_{clutter}=\frac{N_{pre-detect}}{Area} \end{array}$$

### 2.3. Coastal Region Detection

**Figure 12:**
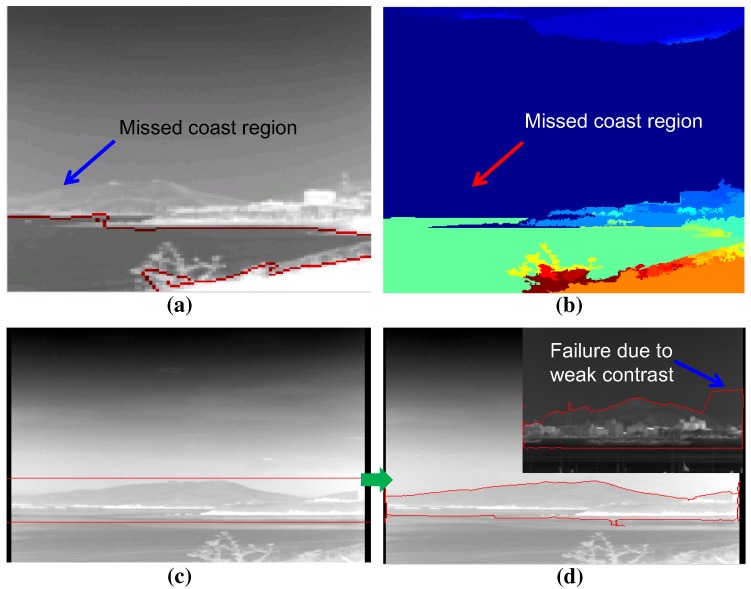
Limitations of the previous region segmentation approaches: (**a**) normalized graph cut (N-cut) with three prior regions; (**b**) mean-shift segmentation; (**c**) prior rectangle for snake; (**d**) after energy minimization in the snake algorithm.

Given the information of the infrared background types, the coastal region should be detected for remote coast and nearest scenes. The state-of-the-art computer vision techniques were applied to find the coastal region in an infrared image. In the bottom-up approach, normalized graph cut (N-cut) is famous for its image segmentation capability [29]. N-cut regards an image as a set of nodes and edges. The strength of the edge increases if the similarity between pixels is strong. If it is not, the edge is cut to separate these pixels. Figure 12a shows the region segmentation result for an infrared image with the number of regions, three. Although the N-cut showed excellent region segmentation in the color image, it showed poor results in the infrared image because of the lack of multi-spectral (red, green, blue) information. Another well-known bottom-up method is the mean-shift segmentation [28]. The mean-shift finds clusters that maximize the local density. Figure 12b presents the application results using the mean-shift algorithm. This works quite well, but it misses the important coastal regions indicated by the arrow. The bottom-up approaches require huge processing time and miss important coastal regions. On the other hand, a top-down region segmentation approach uses prior information of the region of interest, such as the snake or active contour model [30]. The snake moves the initial contours to minimize both the external energy (edge similarity) and internal energy (elastic, bending energy). Figure 12d shows the final region segmentation result using the initial shape shown in Figure 12c. The top-down approach showed higher coastal region detection performance than the bottom-up approaches. On the other hand, it failed when there was weak contrast between the sky and coast region. Furthermore, it required the shape prior, and the initial position of the shape prior was selected manually, which is the weakest point of the algorithm.

**Figure 13:**
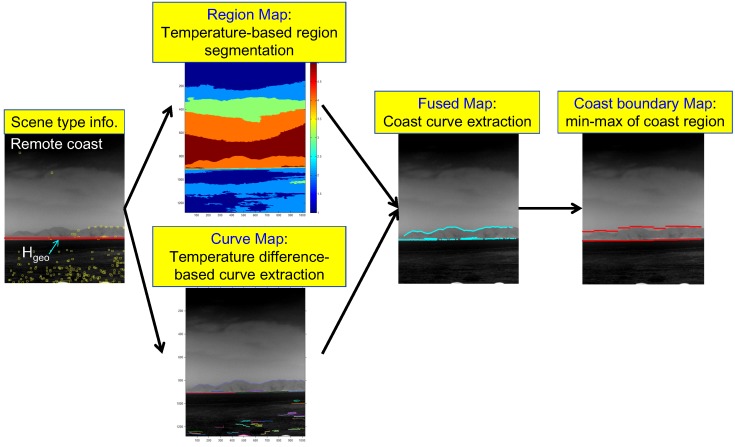
Proposed coast region detection flow given in the scene type information.

A novel coastal region detection method is proposed to overcome the limitations of previous approaches by fusing a region map and a curve map, as shown in Figure 13. A region map is obtained using temperature-based segmentation, and a curve map is obtained using temperature difference-based curve extraction. Two important key issues of coastal region detection are whether or not a coast exists, and if it exists, how to initialize the fusion process to detect the coast region. In this study, the information (scene type, horizon) obtained in the scene type classification block was used. The proposed coast region detector is initiated, if the scene type is determined to be a coastal region (remote, near). After the region map and curve map are extracted, the geometric horizontal line ($H_{geo}$) is used to select the related coast-like region in the region map. The curves that surround the selected coast-like region are then selected. The final coast boundary map is generated by finding the sub-sector-wise local minima and maxima of the fused map.

**Figure 14:**
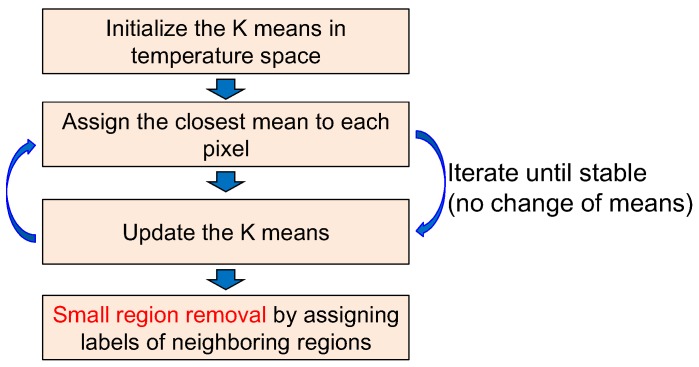
Region map extraction using K-means clustering and small region removal.

**Figure 15:**
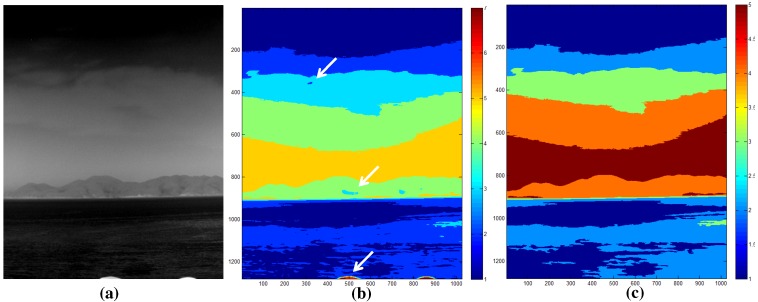
Results of the region map extraction: (**a**) test image; (**b**) original K-means clustering; (**c**) final region map.

A region map ($R_{k}\left(i,j\right)$) is generated using the K-means clustering algorithm, which is useful for segmenting regions according to the thermal energy [31]. *k* represents the region label at the pixel position (i,j). The K-means clustering-based region segmentation method was selected instead of the complex N-cut or mean-shift to reflect the properties of the sky, coast and sea discussed in Section 2.1. The average temperature of the sea region is cold relative to the coast region, despite the sky region having a wide temperature range. Figure 14 shows the K-means clustering algorithm, where K denotes the number of temperature centers. If K is seven, the initial temperature means (digital number) are 0, 36, 72, 108, 144, 180, and 216 for an eight-bit resolution. The label of each pixel is assigned to the label of the closest mean. The iteration continues until convergence, as shown in Figure 15b for the test image of Figure 15a. Furthermore, small region removal by assigning the labels of the neighboring regions is processed to obtain meaningful regions, as shown in Figure 15c. Note that the small isolated regions indicated by the arrows in Figure 15b are removed clearly in Figure 15c.

**Figure 16:**
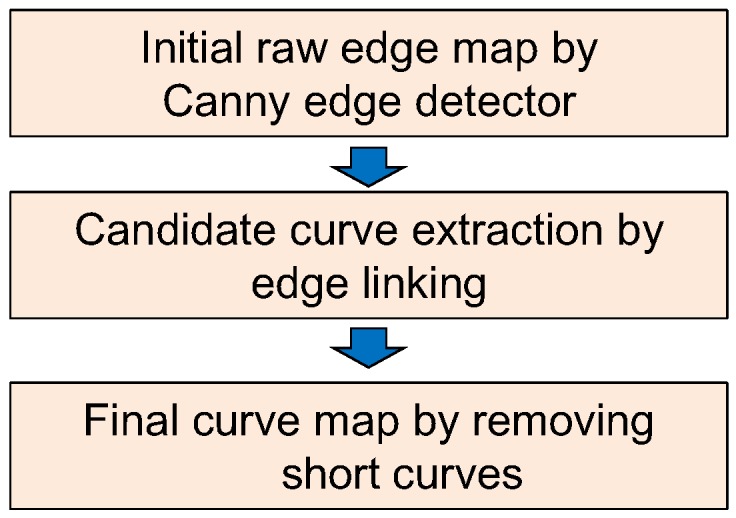
Curve map extraction using Canny edge detection, edge linking and short curve removal.

**Figure 17:**
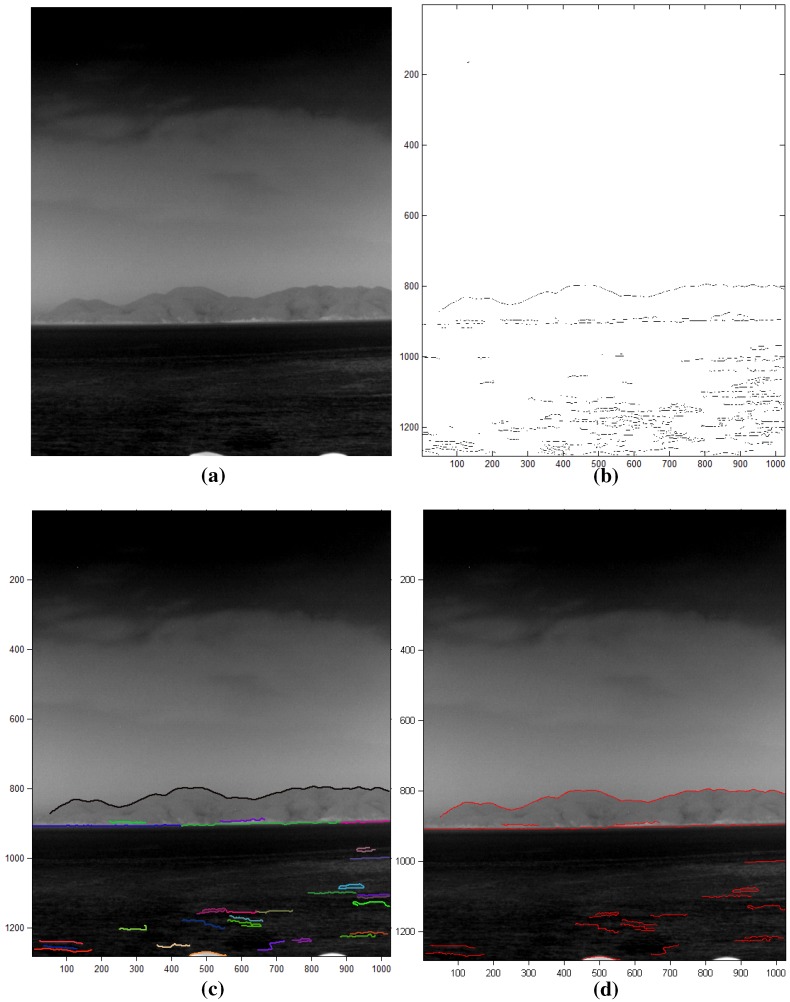
Results of curve map extraction: (**a**) test image; (**b**) initial raw edge map by Canny edge detector; (**c**) contour extraction with a gap size of 2; (**d**) final curve map by removing the short curves.

The sky-coast region and coast-sea region show different temperatures, which cause boundaries in the infrared images. A curve map (C(i,j)) is calculated using the Canny edge detector and edge linking, as shown in Figure 16. The Canny edge detector [25] can determine the temperature differences using the Gaussian derivatives and non-maxima suppression, as shown in Figure 17b for the input image in Figure 17a. The curve fragments are extracted by applying the edge linking method with edge gap filling, as shown in Figure 17c, where different colors represent the curve fragments identified. The final curve map is obtained by limiting the minimum curve length because the sky-coast boundary and coast-sea boundary have a long curve length, as shown in Figure 17d.

**Figure 18:**
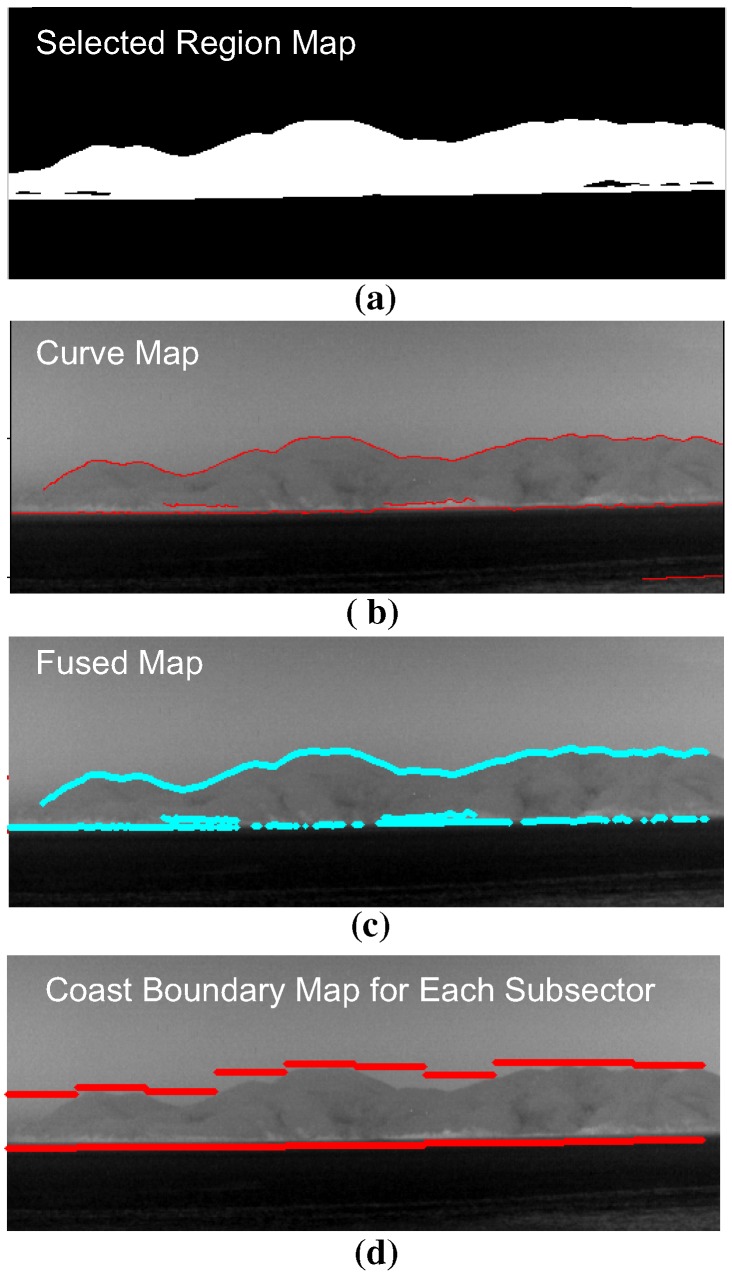
Fused map and coast boundary map generation flow using the selected region map and curve map: (**a**) selected region map using horizon information; (**b**) extracted curve map; (**c**) fused map generation by applying an AND operation to the selected region and curve map; (**d**) coast boundary representation.

**Figure 19:**
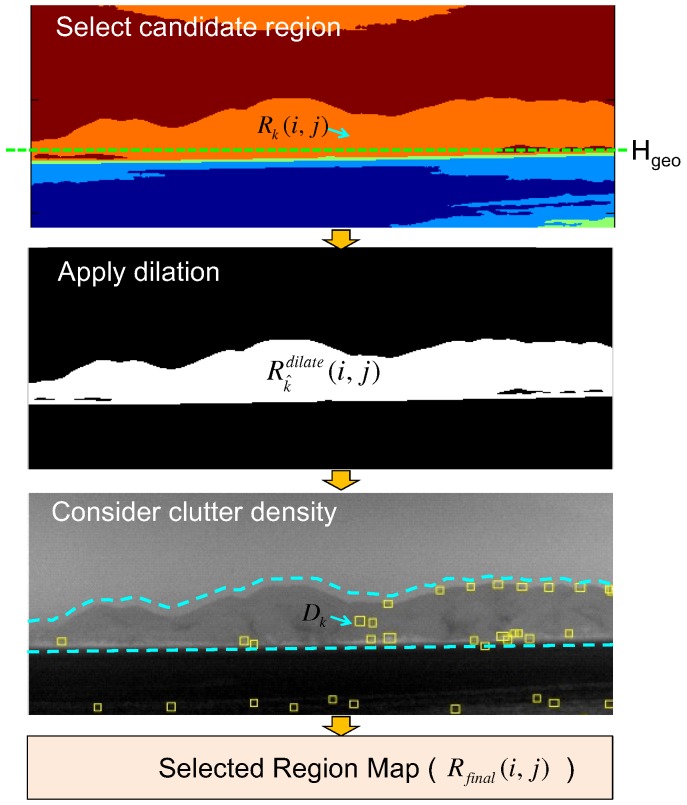
Region map selection flow using the geometric horizon and clutter density.

The fused map (F(i,j)) in Figure 13 can be obtained from the selected region map ($R_{final}\left(i,j\right)$) and curve map (C(i,j)), as shown in Figure 18. The selected region map is the result of the coast-like region selection considering both the geometric horizon line and clutter density, as shown in Figure 19.

The candidate region label set ($\hat{K}$) can be initialized using the predicted geometric horizon ($i=H_{geo}$) along each column position as expressed in Equation (7).

(7) $$\begin{array}{c} \hat{K}=\left\{k|R_{k}\left(i,j\right),i=H_{geo},j=1,2,\cdots ,N\right\} \end{array}$$

The region ($R_{\hat{k}}\left(i,j\right)$) with a specific label ($\hat{k}\in \hat{K}$) is expanded using a morphological dilation method [32] to include the coastal edges using Equation (8).

(8) $$\begin{array}{c} R_{\hat{k}}^{dilate}\left(i,j\right)=Dilate\left(R_{\hat{k}}\left(i,j\right)\right) \end{array}$$

The initial region labels are refined further (${\hat{K}}_{D}$) using the density ($D_{\hat{k}}$) of clutter of the related regions, as expressed in Equation (9). The clutter density of a region with an index of $\hat{k}$ can be estimated by dividing the number of pre-detections ($N_{pre-detect}\left(R_{\hat{k}}\right)$) by the area of the specific region ($R_{\hat{k}}$), as expressed in Equation (10).

(9) $$\begin{array}{c} {\hat{K}}_{D}=\left\{\hat{k}|D_{\hat{k}}>\epsilon ,\hat{k}\in \hat{K}\right\} \end{array}$$

(10) $$\begin{array}{c} D_{\hat{k}}=\frac{N_{pre-detect}\left(R_{\hat{k}}\right)}{Area(R_{\hat{k}})} \end{array}$$

The finally selected region map ($R_{final}\left(i,j\right)$) is generated by merging the surviving coast-like regions, as expressed in Equation (11).

(11) $$\begin{array}{c} R_{final}\left(i,j\right)=\sum_{\hat{k}\in {\hat{K}}_{D}}R_{\hat{k}}^{dilate}\left(i,j\right) \end{array}$$

The fused map (F(i,j)) is obtained by applying AND fusion [33] of the selected region map ($R_{final}\left(i,j\right)$) with the curve map (C(i,j)), as expressed in Equation (12).

(12) $$\begin{array}{c} F\left(i,j\right)=R_{final}\left(i,j\right)\cap C\left(i,j\right) \end{array}$$

## 3. Experimental Results

**Figure 20:**
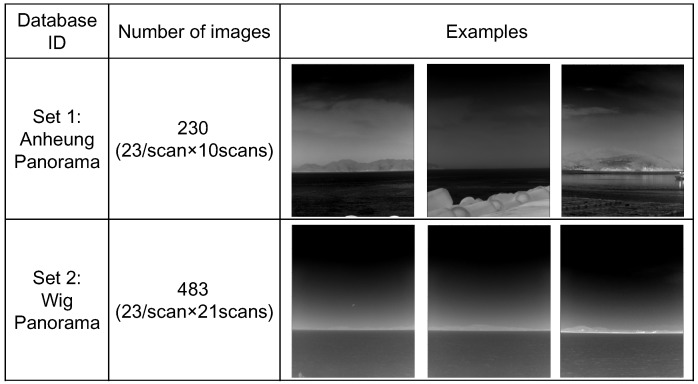
Composition of the infrared database.

Panoramic IRST images were prepared to validate the proposed method, as shown in Figure 20. One panoramic image consisted of 23 sector images, where one sector image covered a field of view of $15^{\circ }$. In addition, each sector image had an image resolution of 1280×1024 and contains sky-sea, sky-coast-sea or sky-coast, depending on the viewing direction.

**Figure 21:**
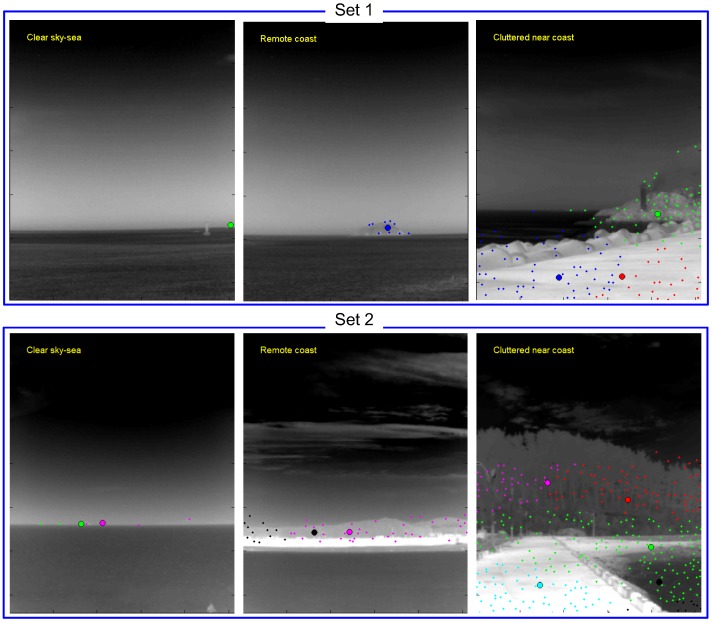
Examples of successful infrared scene type classification.

In the first experiment, the proposed infrared scene type classifier was evaluated for the two test sets. Table 1 lists the overall classification performance for each set. Three scene types (sky-sea, cluttered remote coast, cluttered near coast) were considered, because there were no related scenes with a homogeneous near coast, even though the proposed algorithm can classify them. The overall classification accuracy was approximately 95.6% for this database. Several miss-classifications occurred for the cluttered remote coast where a few pre-detections existed (classified as sky-sea). Figure 21 gives partial examples of the infrared scene type classification using the proposed horizon information and pre-detection results.

**Table 1:** Infrared scene type classification performance for the test database using the proposed horizon and clutter density-based method.

Dataset	Sky-Sea	Cluttered Remote Coast	Cluttered Near Coast	Accuracy (%)
Set 1	60/60	60/60	100/110	95.6% (220/230)
Set 2	42/42	294/315	126/126	95.7% (463/484)

**Figure 22:**
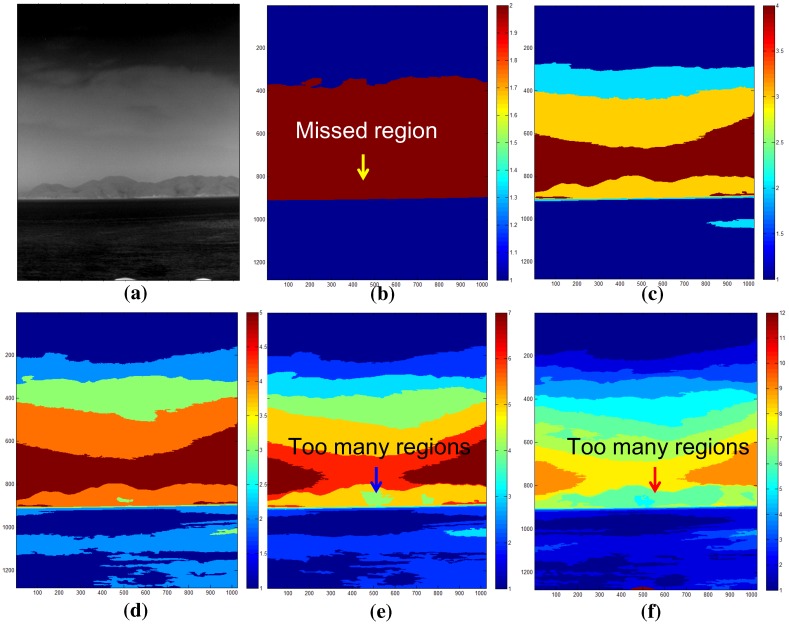
The effects of the parameter K in the K-means segmentation: (**a**) test image; (**b**) K = 3; (**c**) K = 5; (**d**) K = 7; (**e**) K = 9; and (**f**) K = 12.

In the second experiment, the effect of the K value in the K-means segmentation was evaluated. The control parameter K is one of the most important parts in the proposed method. It can control the number of segmented regions and should be selected depending on the scenario. Figure 22 shows the effects of thermal image segmentation depending on the control parameter K. For a test image (Figure 22a), K with two cannot segment the coast region, as shown in Figure 22b. K with five or seven can segment it correctly (Figure 22c,d). However, K values larger than seven produce too many regions, which is not suitable for the proposed coast detection method, as shown in Figure 22e,f. Therefore, the optimal K values are five or seven, depending on the thermal image environments.

In the third experiment, the performance of the coastal region detection was evaluated for the test set. The mean-shift segmentation method [28] was used as a baseline method because it is used widely and has shown excellent performance in the computer vision community. Geometric horizon information-based coastal region selection was inserted after the original mean-shift segmentation to identify the coastal region. The infrared scene type information was assumed to have been given for both the baseline method and the proposed method. Therefore, the coastal region detection methods are activated if the scene type contains a coastal region. The coastal region detection rate is quite difficult to measure. In this study, a sub-sectional image-wise region overlap measure was used, as expressed in Equation (13) [34]. $R_{t}$ and $R_{r}$ represent the detected coastal region and ground truth coastal region, respectively. Two regions were determined to correspond if the region overlap was large enough (>0.5).

(13) $$\begin{array}{c} \mathrm{correct}\;\mathrm{detection}\;\mathrm{if}\;\frac{R_{t}\cap R_{r}}{R_{t}\cup R_{r}}>0.5 \end{array}$$

**Figure 23:**
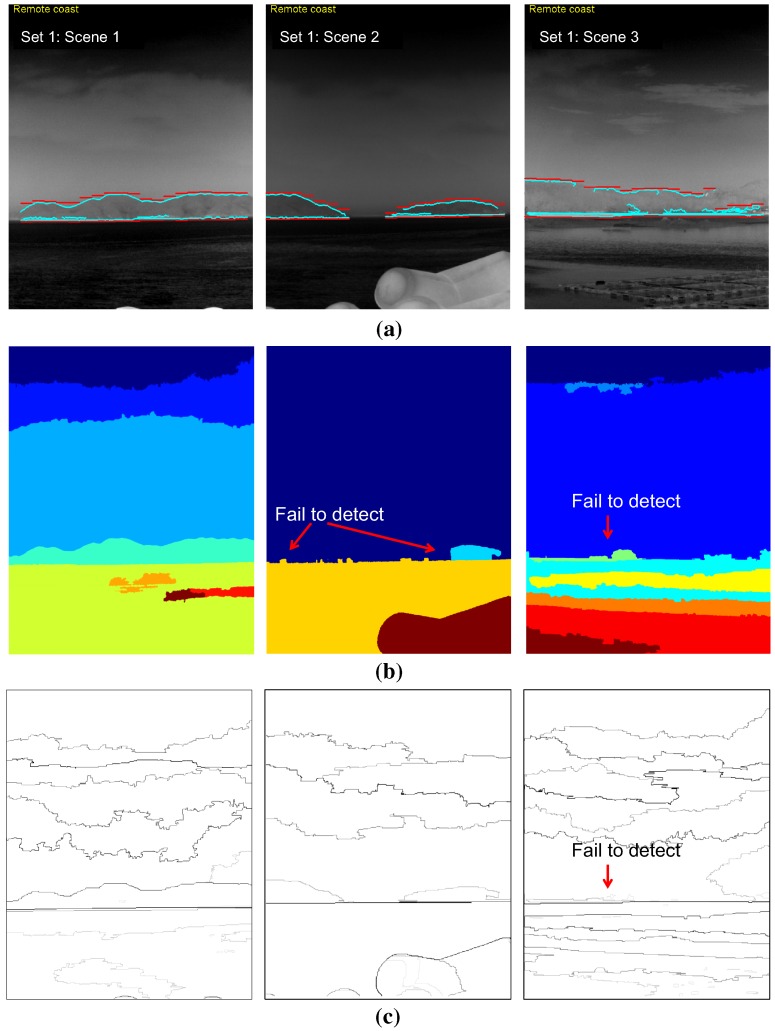
Comparison of the coastal region detection results for Test Set 1: (**a**) proposed coastal region detection; (**b**) mean-shift segmentation-based method [28]: Scene 1 was successful; Scenes 2 and 3 failed (segmentation results were displayed as partial outputs.) [28]; (**c**) statistical region merging method [35].

**Figure 24:**
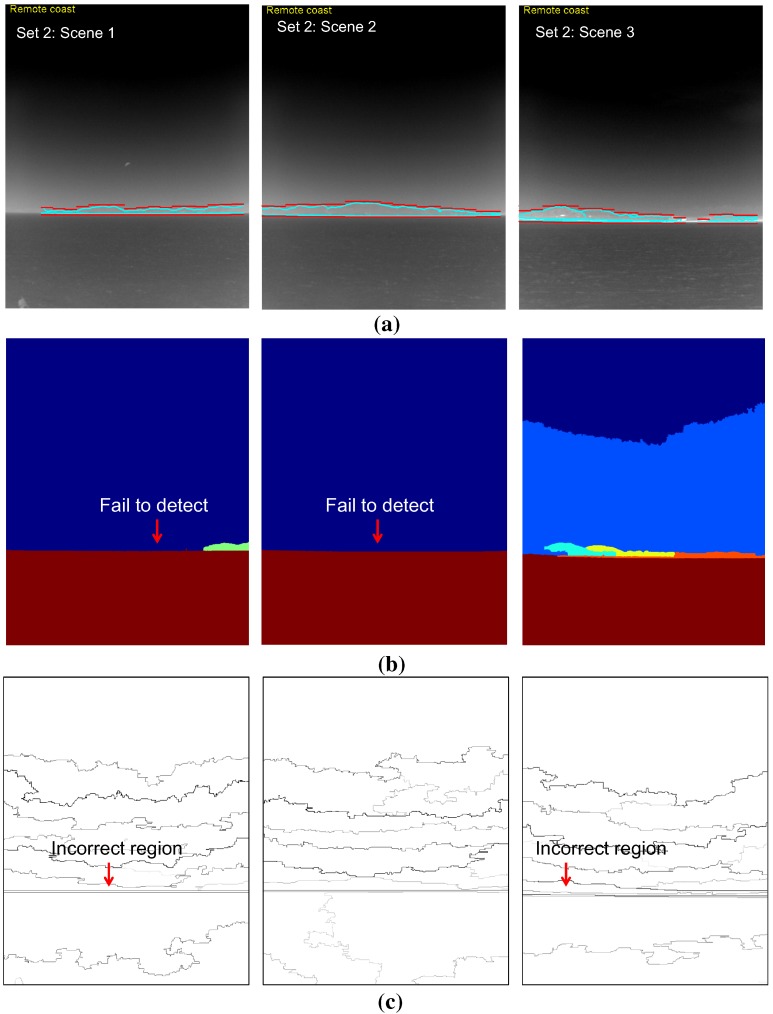
Comparison of the coastal region detection results for Test Set 2: (**a**) proposed coastal region detection; (**b**) mean-shift segmentation [28]: it failed for these test scenes; (**c**) statistical region merging method [35].

Table 2 lists the results of a quantitative evaluation of the coastal region detection using the proposed method and two baseline methods (mean-shift segmentation [28] and statistical region merging [35]) for the selected sky-coast-sea images. The proposed method outperformed (overall detection rate: 96.4%) the well-known mean-shift segmentation (overall detection rate: 34.8%) and statistical region merging (overall detection rate: 66.%). The parameters of the mean-shift segmentation were tuned to identify the coastal regions (spatial band width: 7, range band width: 7, minimum region area: 5000, gradient window radius: 7, edge strength threshold: 1). Those of the statistical region merging were set to the original method. Figure 23 and Figure 24 show the corresponding coastal region detection results for both methods. The proposed method failed on the right three consecutive sub-sectors in the Set 1:Scene 3 image, where the sky-coast line is ambiguous. The mean-shift segmentation was found in the coastal regions on the Set 1-Scene 1 with image segmentation and geometric horizon information. On the other hand, it failed on the remaining test images, because it could not segment out the ambiguous coast regions. In the quantitative evaluation, partial segmentation results of the coastal regions were regarded as a success for the failure cases. The strength of the contour represents the belief of the segmented region in statistical region merging method. The coastal regions can be found quite well compared to the mean-shift segmentation. On the other hand, it failed to find the coast regions in ambiguous sky-coast area.

**Table 2:** Infrared coastal region detection performance for the test database using the proposed method and baseline method (mean-shift segmentation) [28]. DR denotes detection rate. $N_{d}$ and $N_{t}$ represent the number of detections and the number of ground truths, respectively.

Test scene	Proposed		Mean-Shift Segmentation [28]		Statistical Region Mergin [35]	
	$N_{d}/N_{t}$	DR [%]	$N_{d}/N_{t}$	DR [%]	$N_{d}/N_{t}$	DR [%]
Set 1:Scene 1	19/19	100%	19/19	100%	19/19	100%
Set 1:Scene 2	19/19	100%	2/19	10.5%	19/19	100%
Set 1:Scene 3	16/19	84.2%	0/19	0%	0/19	0%
Set 2:Scene 1	17/17	100%	2/17	11.8%	0/19	0%
Set 2:Scene 2	19/19	100%	0/19	0%	19/19	100%
Set 2:Scene 3	18/19	94.7%	16/19	84.2%	17/19	89.4%
Overall	108/112	96.4%	39/112	34.8%	74/112	66.0%

**Figure 25:**
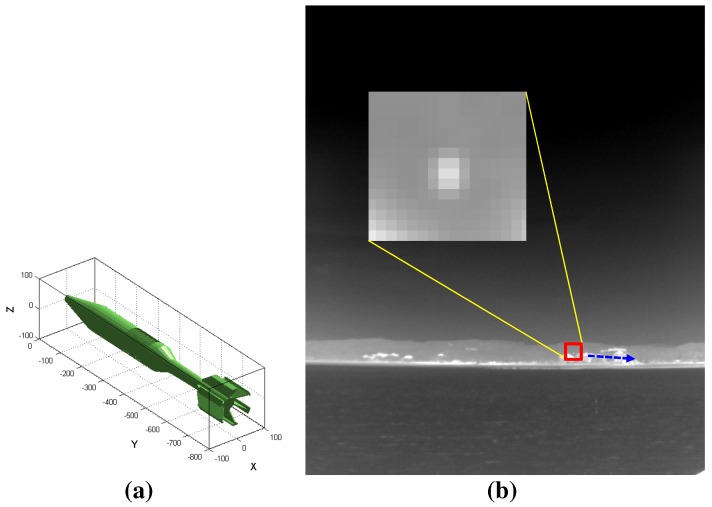
Generation of synthetic test images by inserting a 3D CAD model into the coast background image [36]: (**a**) 3D CAD model of a missile; (**b**) generated infrared image with the target motion.

**Figure 26:**
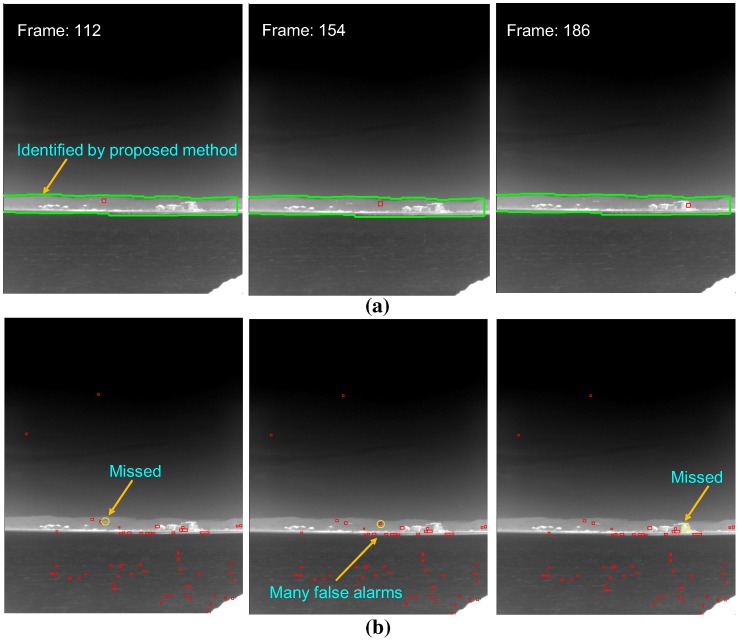
Effect of the coast region information in the infrared small target detection problem for the synthetic DB: (**a**) application of the temporal filter-based detector (TCF) for the identified coast region; (**b**) application of a spatial filter-based detector (top-hat). The yellow circles denote the ground truths, and the red rectangles represent the detection results.

**Figure 27:**
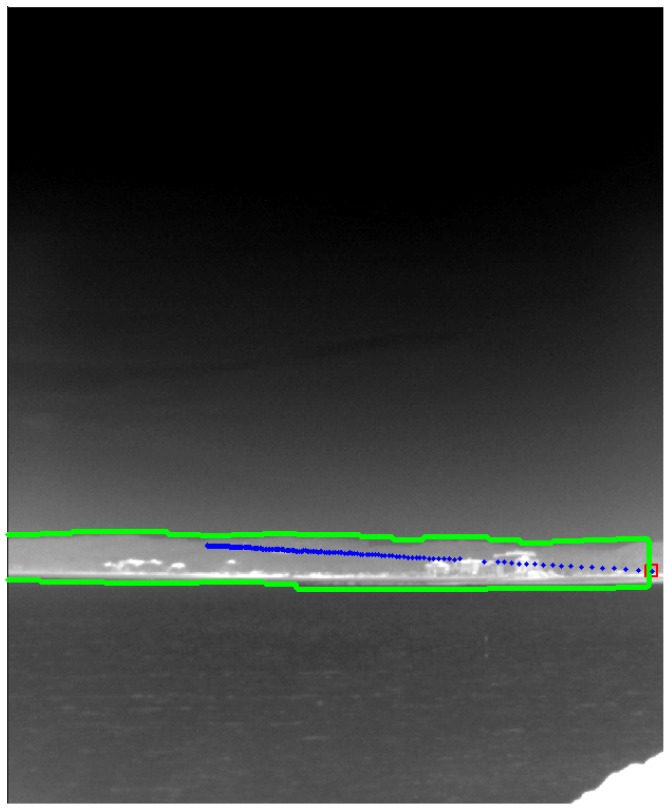
Final footprint of target detection using a temporal filter (TCF) in the identified coastal region. The blue dots represent the detected target locations.

**Figure 28:**
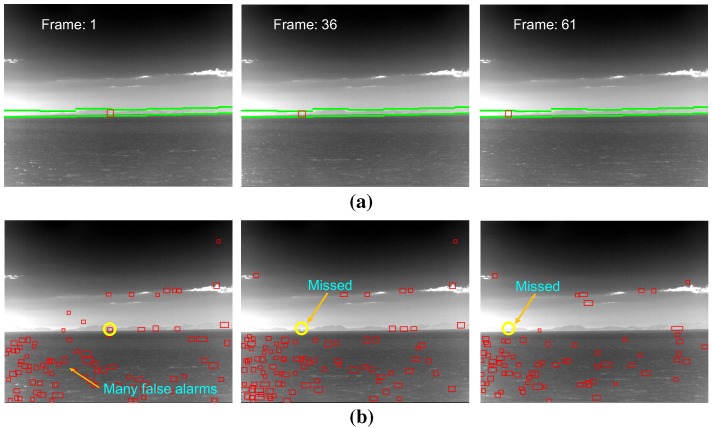
Effect of the coast region information in the infrared small target detection problem for the real WIG craft DB: (**a**) application of the temporal filter-based detector (TCF) for the identified coast region; (**b**) application of spatial filter-based detector (top-hat). The yellow circles denote the ground truths, and the red rectangles represent the detection results.

As a final experiment, the effects of the coastal region information were evaluated by applying small infrared target detection methods to the synthetic infrared test images and real WIGcraft sequence. The synthetic images were generated by inserting a 3D CAD model into the coast background [36], and the real WIG craft sequence was acquired using a CEDIPMWIRcamera. Figure 25 gives an example of the test sequence, where a synthetic target moves around a cluttered coast region. The target radiation was set to 150 W/sr, and the target moves along the coast region with a velocity of Mach 3 at a 2-km distance (target yaw angle $2^{\circ }$, pitch angle $0.38^{\circ }$). The target detection scheme can be selected if the information on the coastal region is available. Normally, a spatial filter-based detector, such as top-hat [5], should be applied around the sky-sea horizontal region to detect the distant targets (almost stationary and dim). On the other hand, the proposed method can provide the scene type and the coast region information if the scene type is classified as the coast background. Because the coast regions are normally located near the sensor position, the target motion can be detected easily using the temporal filter-based method, such as the temporal contrast filter (TCF) [37]. Table 3 lists the target detection performance depending on the coast information for the two types of databases. The detection rate by applying TCF to the recognized coast region method was better than that of top-hat, where the coast information was unavailable. Furthermore, the false alarm rate (FAR) of the coast information-based method was reduced to zero/image. Figure 26 shows the related partial detection results depending on the coastal region information. Figure 27 presents the final footprints of target detection in the coastal region. Figure 28 shows the WIG craft detection results with the same test environments. Through the evaluation, the proposed coast region information can upgrade the target detection performance in the sea-based IRST problem.

**Table 3:** Infrared coastal region detection performance for the test database using the proposed method and the baseline method (mean-shift segmentation) [28]. FAR denotes the false alarm rate. TCF, temporal contrast filter.

DB Types	Performance Measure	With Coast Information by the Proposed Method Temporal Filter (TCF [37])	Without Coast Information Spatial Filter (Top-Hat [5])
Synthetic	Detection rate	**97.7**% (171/175)	89.7% (157/175)
DB	FAR	**0**/image	54/image
WIGcraft	Detection rate	**98.3**% (60/61)	85.3% (52/61)
DB	FAR	**0**/image	65/image

**Figure 29:**
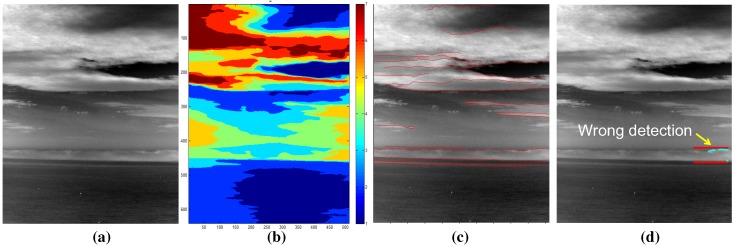
Examples of a failure case: (**a**) dense cloud clutter around horizon and sky; (**b**) extracted region map; (**c**) extracted edge map; (**d**) coast detection using the proposed method.

As validated in previous experiments, the proposed method works for a normal sky-coast-sea background. The proposed method produces partly false coast detection if there is a large portion of clouds in the sky and the clouds are close to the horizon, as shown in Figure 29. In this case, the false region map and cloud edge map lead to erroneous results.

## 4. Conclusions and Discussion

Extracting the coast region is important for the successful operation of infrared search and track. This paper proposed a two-stage-based infrared scene interpretation by the consecutive scene type classification and coastal region detection. The coastal region detector was designed to be activated when the infrared scene contains the coast background. In the scene type classifier, the sea-based infrared background has no distinctive texture features according to gray level co-occurrence matrix (GLCM) analysis. On the other hand, the relationship between the geometrically-predicted horizon and image horizon provides a clue to the existence of a coastal region. In addition, the clutter density by pre-detection provides an additional clue to the clutter type of observed coast regions. The final coastal region detector is activated when the probing scene contains a near coast or remote coast. State-of-the-art computer vision techniques, such as normalized cut (N-cut) or mean-shift segmentation, showed poor region segmentation results due to the ambiguous sky-coast regions. This paper proposes a fusion-based method using a region map and a curve map. The region map was generated by K-means-based temperature segmentation, and the curve map was generated by a Canny edge detector and edge linking. The coast region was initiated by the geometric horizon information and confirmed whether the surrounding curves exist. The proposed method was evaluated by three experiments: scene type classification performance, coastal region detection performance and the effect of the coast region information on infrared small target detection. Various experiments on the real and synthetic infrared image showed convincing results. In the first evaluation, the proposed scene type classifier showed a classification accuracy of 95.7% for hundreds of test images. In the second evaluation, the proposed coastal region detection showed a detection rate of 96.4%, and the baseline method (mean-shift segmentation) showed a detection rate of 34.8%. In the final evaluation, the coastal region information enhanced the target detection rate and reduced the false alarm rate by applying a temporal filter-based method for the identified coast region. In the future, the test will be conducted on more and varied IRST operation scenarios.
